# User Experience and Usability of Neumorphism and Gamification User Interface Designs in an HIV Self-Test Referral Program for Men Who Have Sex With Men: Prospective Open-Label Parallel-Group Randomized Controlled Trial

**DOI:** 10.2196/35869

**Published:** 2022-06-22

**Authors:** Tsz Ho Kwan, Denise Pui Chung Chan, Shui Shan Lee

**Affiliations:** 1 Stanley Ho Centre for Emerging Infectious Diseases The Chinese University of Hong Kong Hong Kong China (Hong Kong)

**Keywords:** HIV, self-test, men who have sex with men, gamification, neumorphism, digital intervention, HIV prevention, user interface, games, digital health

## Abstract

**Background:**

Digital interventions have been applied for promoting HIV prevention and care among men who have sex with men (MSM). As user interface (UI) design plays a role in determining usability and user experience (UX), the intervention outcome could be affected.

**Objective:**

In this study, we hypothesized that 2 UI design styles, namely gamification and neumorphism, could impact usability and be differentially preferred by distinct groups of MSM.

**Methods:**

A prospective parallel-group open-label randomized controlled trial was conducted in Hong Kong. Eligible participants were adult MSM recruited by the research team or referred by enrolled participants, who followed instructions for performing an HIV self-test and promoted its use within their social network. Participants were randomized in a 1:1 ratio into either a gamification or neumorphism arm, with primarily visual differences in the UI only. The primary outcome was usability measured by the System Usability Scale (SUS) between the 2 arms. Distinct characteristics of promoters in the 2 arms who gave an SUS score of 80 or above were identified.

**Results:**

Of 463 MSM registered in the study, 232 and 231 were randomized to the gamification and neumorphism arms, respectively. Excluding those who did not request a self-test kit, data from 218 and 216 participants in the gamification and neumorphism arms, respectively, were analyzed (totally 434 participants). With a median SUS score of 80 overall, participants in the neumorphism arm gave a higher score (*P*<.001), with a higher proportion giving a promoter-level SUS score (*P*=.002). Promoters used social media for sex networking (*P*=.02), used pre-exposure prophylaxis in the preceding year (*P*=.006), had higher satisfaction in UI design (*P*<.001), and had made a self-test referral (*P*=.04). In general, higher usability was recorded among participants who were confident in performing the HIV self-test (*P*<.001), and this was associated with a promoter-level SUS score in both arms. While no other personal characteristics were associated with promoters in the neumorphism arm, those in the gamification arm had higher HIV-related knowledge (*P*=.01), preferred a specific partner body image type (*P*=.03), and progressed toward peer referral by completing online training (*P*=.04).

**Conclusions:**

Both gamified and neumorphic UI designs were well-accepted by MSM. UX and satisfaction of UI were both crucial in influencing the willingness of MSM to promote the application by referring their peers in the community to participate. The simplistic visual design of neumorphism conferred a more general acceptance in the community, whereas gamification was preferred in certain MSM subcommunities. Appropriate UI/UX design should be considered when developing digital interventions targeting the MSM community.

**Trial Registration:**

ClinicalTrials.gov NCT04379206; https://clinicaltrials.gov/ct2/show/NCT04379206

## Introduction

Digital technology has been leveraged as an innovative approach to improve health in different settings, especially in HIV care and prevention. It was adopted for promoting HIV testing through the use of specially designed apps targeting the men who have sex with men (MSM) community [1], monitoring adherence of medication intake [2], and providing HIV prevention materials to the community [3]. A qualitative study showed that ease of operation is an important factor for MSM to use and keep using such apps [4]. This is in line with concepts of the theoretical framework in the technology acceptance model stating that perceived ease of use and usefulness contribute to one’s intention to use technology [5]. Perception of ease of use is underpinned by user interface (UI) design, such that poor visual design could negatively affect the user experience (UX) and create usage challenges [6]. Therefore, UI design is one of the key elements influencing the usability of a digital intervention [7].

There are many models and styles in UI design, and a popular one is gamification. Gamification is the application of video game elements in nongaming contexts to enhance UX [8]. It has been applied for improving drug adherence in rheumatoid arthritis patients [9] and for supporting clinical decision-making [10]. The effectiveness of gamification has been shown in the promotion of health among MSM. A previous study found that a gamified mobile app could improve HIV knowledge and confidence in adherence to HIV medications among these individuals [11]. The positive effect of gamification was markedly higher in regular users [12]. With rewards like badges, credit points, and actual prizes, and social elements, such as leader boards, the elements in the gamified experience were not just acceptable to MSM, but could also motivate them to engage in promotional activities [13]. On the other hand, a new UI design style, neumorphism or soft UI, emerged in late 2019, which was introduced by Alexander Plyuto [14]. It is a simplistic version of skeuomorphism, which was widely used in the past to imitate a real-world physical object in the UI. Neumorphism simplifies skeuomorphism by making use of light and dark shadows to accentuate the UI element from its background with the same color while capturing the realistic form of the object [15]. The combination of realistic element and the simplistic design can offer an interactive and tactile UX [16].

The 2 UI design styles of gamification and neumorphism were visually distinctive, leading us to hypothesize that different groups of MSM may be in favor of either design. Against these backgrounds, we aimed to assess the differential impact of gamification and neumorphism UI design styles on UX and usability in a web application developed for promoting HIV self-testing among MSM.

## Methods

### Study Design and Participants

This was a prospective parallel-group open-label randomized controlled study. The study was conducted in Hong Kong, where MSM accounted for a majority of new HIV infections in the past decade [17]. Through a social network approach, enrolled eligible participants were men who ever had sex with other men, who were aged 18 years or above, and who were either referred by another participant in the study or recruited by the research team as seeds. The exclusion criterion was inability to communicate in written English or Chinese. Block randomization was adopted with a block size of 4 and an allocation ratio of 1:1, using a computer-generated list concealed in server-side codes in the web application. With a sample size of 400, a mean difference in the acceptance score of 1% could be detected at an assumed standard deviation of 10% between 2 arms.

### Intervention

**Figure 1:**
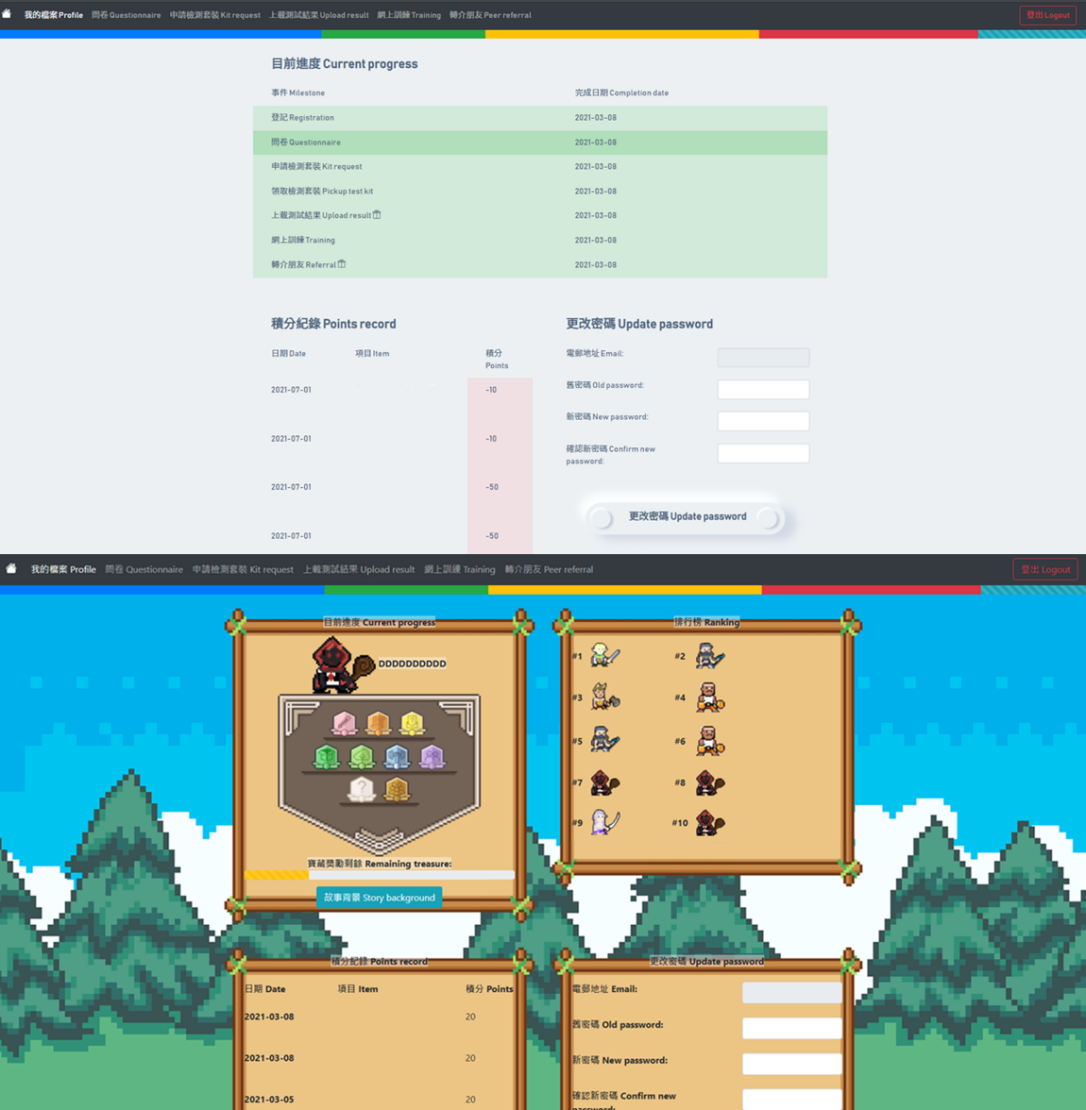
The profile pages of the neumorphism (upper panel) and gamification (lower panel) designs.

The participants were randomized into either the neumorphism or gamification UI arm, with the unified objective of undertaking a free HIV self-test using oral fluid or blood at their will. The differences in the 2 arms were related to the UI only (primarily visual). In the neumorphism arm, in terms of form elements, the buttons and frames were in the neumorphism style, while checkboxes and radio buttons remained browser default to balance readability and identification of form elements (Multimedia Appendix 1). A monochromatic color scheme was adopted in compliance with the neumorphism design style. To enrich a gamified UX, the process table in the neumorphic design was replaced by a badge board, and a story background was created (Figure 1). Each of the pages in the gamification arm had a unique backdrop simulating different places in a role-playing game. The profile and questionnaire pages appeared as a forest in daytime and at night, respectively. The test kit request and result upload pages depicted a sword to be picked from the soil and items to be devoted in a shrine, respectively. While training was illustrated as a magical book, the backdrop on the referral page was a tavern. The incentive redemption page was assimilated as a reward bestowed by the “crown.” The study procedures remained the same between the 2 arms, namely the questionnaire, test kit request, test result upload, online training, and peer referral.

### Data Collection

The questionnaire contained questions on demographics; sex networking characteristics, including self-perceived body image types and preferences of sex partners’ body image types [18,19], history of sexual behavior, sex networking, and HIV testing; preferences of self-tests; and 17 questions on HIV-related knowledge. The primary outcome of the study was the usability of the web application measured by the System Usability Scale (SUS) [20] and the Single Ease Question (SEQ) [21], as well as the satisfaction of the UI designs of the 2 styles. The SEQ was used after each task throughout the study, whereas the SUS was only displayed after test result upload and peer referral. Participants were free to fill out these scales without obstruction to the main study procedure. Akin to the SUS, an additional question, “I am satisfied with the system interface design,” with a scale ranging from 1 (strongly disagree) to 5 (strongly agree), was used to assess participants’ acceptance of the UI (UI score). For individual SUS items, a score of at least 4 out of 5 was considered a positive response, whereas for the SEQ, a score of 5 or higher on the scale of 7 was regarded as positive. Overall, the SUS score was calculated according to the standard by inversing responses in even number questions and then multiplying the scores by 2.5. Previous research has shown that an SUS score of 68 is average [22], 71 is acceptable [23] or “good” [24], and 80 indicates a user is willing to promote the product or system to a friend [21]. In the analysis, we adopted the latter 2 thresholds as the definitions of acceptance and participation as a promoter of the system, respectively. Two subscale scores were derived from the SUS by separating items 4 and 10 from the remainder to form the learnability and usability scores, respectively, and the same threshold was used.

### Analysis

The scores of the UI, individual SUS items, SUS main scale and subscales, and SEQ at different stages were compared between the 2 arms, and other participant attributes and sex networking characteristics were compared using the Mann-Whitney *U* test. With an overarching aim to examine the willingness of referring peers to undergo self-testing, the SUS scores were dichotomized by the promoter-level threshold, and associated factors were identified per arm by univariable and multivariable logistic regression models. The analyses and reporting of the results followed the CONSORT-eHEALTH (Consolidated Standards of Reporting Trials of Electronic and Mobile Health Applications and Online Telehealth) guidelines [25] (Multimedia Appendix 2). This study has been registered at ClinicalTrials.gov (ID: NCT04379206). All analyses were conducted in R software (R Project for Statistical Computing).

### Ethics Approval

This study was approved by the Joint Chinese University of Hong Kong – New Territories East Cluster Clinical Research Ethics Committee and the Ethics Committee of the Department of Health (CREC reference number 2020.087).

## Results

**Figure 2:**
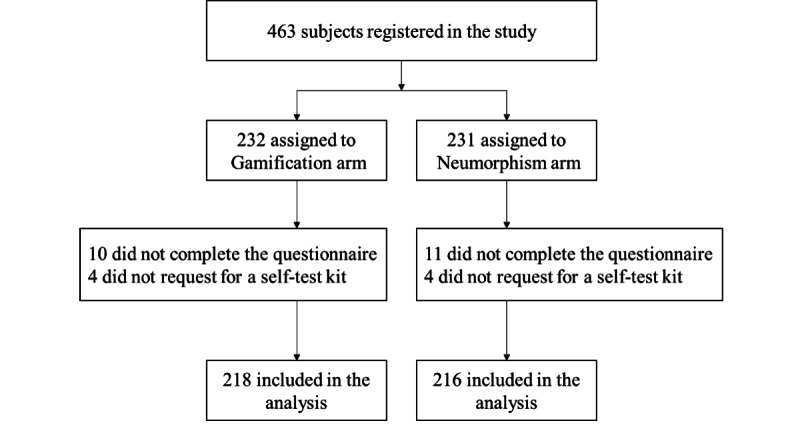
Study flow.

Overall, 463 MSM registered in the study between March 1, 2021, and May 12, 2021, of whom, 232 and 231 were assigned to the gamification and neumorphism arms, respectively (Figure 2). After excluding 21 subjects who had not completed the questionnaire and 8 who failed to request a self-test kit, data from 434 participants were analyzed. Overall, the participants’ median age was 28 years (IQR 24-33 years) (Table 1). Moreover, 76.5% (332/434) and 43.3% (188/434) of participants had used location-based MSM social networking apps and social media apps, respectively, to seek male sex partners in the past year, whereas patronizing physical venues, such as bars (33/434, 7.6%), saunas (56/434, 12.9%), and sex parties (26/434, 6.0%), for sex networking amidst the COVID-19 pandemic when social distancing policies were in force was rare. In terms of HIV-related knowledge, 59.0% (256/434) of participants got 11 correct answers and 21.0% (91/434) gave at the most 3 wrong answers out of 17 question items. Overall, 340 (78.3%) had tested for HIV before, of whom, 139 (40.9%) never used an HIV self-test product. More participants were confident to collect sufficient oral fluid (269/434, 62.0%) than blood (197/434, 45.4%) for the self-test. In addition, 66.8% (290/434) were confident in performing self-tests and 70.3% (305/434) were confident in interpreting the test results. A similar proportion (282/434, 65.0%) did not prefer assistance in self-tests.

**Table 1:** Participants’ characteristics.

Characteristic		Overall (N=434)	Gamification arm (N=218)	Neumorphism arm (N=216)
Age (years), median (IQR)		28 (24-33)	28 (24-32)	29 (24-34)
**Sex networking in the past 1 year,** **n (%)**				
	Used location-based MSM^a^ social networking apps	332 (76.5)	163 (74.8)	169 (78.2)
	Used social media apps	188 (43.3)	90 (41.3)	98 (45.4)
	Patronized local bars	33 (7.6)	18 (8.3)	15 (6.9)
	Patronized local saunas	56 (12.9)	35 (16.1)	21 (9.7)
	Attended local sex parties	26 (6.0)	18 (8.3)	8 (3.7)
Gave a correct answer in at least 11 out of 17 questions on HIV-related knowledge, n (%)		256 (59.0)	130 (59.6)	126 (58.3)
Gave a correct answer in at least 14 out of 17 questions on HIV-related knowledge, n (%)		91 (21.0)	46 (21.1)	45 (20.8)
Ever tested for HIV, n (%)		340 (78.3)	166 (76.1)	174 (80.6)
Ever HIV self-tested (N=340), n (%)		201 (59.1)	101 (60.8)	100 (57.5)
**Confidence in self-test procedures, n (%)**				
	Collecting sufficient oral fluid^b^	269 (62.0)	135 (61.9)	134 (62.0)
	Collecting sufficient blood^b^	197 (45.4)	90 (41.3)	107 (49.5)
	Correctly performing the HIV self-test^b^	290 (66.8)	142 (65.1)	148 (68.5)
	Interpreting the self-test result^b^	305 (70.3)	146 (67.0)	159 (73.6)
	Preferring no assistance for the self-test	282 (65.0)	143 (65.6)	139 (64.4)

^a^MSM: men who have sex with men.

^b^Gave a score of 9 or above on a scale of 0 to 10.

The median SUS score given at test result upload was 80.0 (IQR 70.0-90.0), with median learnability and usability scores of 87.5 (IQR 62.5-100.0) and 78.1 (IQR 68.8-90.6), respectively. Higher learnability was scored by MSM who used social media for sex networking (*P*=.02), preferred sex partners to be of the “cute” body image type (*P*=.009), never had sex with a female individual (*P*=.04), and gave more correct answers in HIV-related knowledge (*P*=.005) (Table 2). They also gave higher confidence scores in sample collection for both oral fluid (*P*=.002) and blood (*P*=.004), performance of the self-test (*P*<.001), and result interpretation (*P*<.001). A higher usability score was given by participants who used social media for sex networking (*P*=.002), preferred sex partners with the body image type of “cute” (*P*=.03) or “decent” (*P*=.02) but not “mature” (*P*=.04), were confident in using the self-test (*P*<.01), and returned the self-test result within 24 hours upon receiving the self-test kit (*P*=.04) (Table 3).

**Table 2:** Differential patterns of learnability scores and associated participant characteristics (N=351).

Variable			Learnability score when the variable is false, median (IQR)		Learnability score when the variable is true, median (IQR)		*P* value
Used social media for sex networking in the past 1 year			87.5 (62.5-100.0)		87.5 (75.0-100.0)		.02
Preferred sex partners to be of the “cute” body image type			87.5 (62.5-100.0)		87.5 (75.0-100.0)		.009
Ever had sex with a female individual			87.5 (75.0-100.0)		75.0 (62.5-100.0)		.04
Gave at least 14 correct answers out of 17 questions on HIV-related knowledge			87.5 (62.5-100.0)		87.5 (75.0-100.0)		.004
**Confidence in self-test procedures**							
	Collecting sufficient oral fluid^a^	75.0 (62.5-100.0)		87.5 (75.0-100.0)		.002	
	Collecting sufficient blood^a^	75.0 (62.5-100.0)		87.5 (75.0-100.0)		<.001	
	Correctly performing the HIV self-test^a^	75.0 (62.5-100.0)		87.5 (75.0-100.0)		<.001	
	Interpreting the self-test result^a^	75.0 (62.5-87.5)		87.5 (75.0-100.0)		<.001	
	Preferring no assistance for the self-test	75.0 (50.0-100.0)		87.5 (75.0-100.0)		<.001	

^a^Gave a score of 9 or above on a scale of 0 to 10.

**Table 3:** Differential patterns of usability scores and associated participant characteristics (N=351).

Variable			Usability score when the variable is false, median (IQR)		Usability score when the variable is true, median (IQR)		*P* value
Used social media for sex networking in the past 1 year			75.0 (68.8-87.5)		84.4 (71.9-93.8)		.002
**Preferred body image type of sex partners**							
	“Cute” type	78.1 (68.8-90.6)		81.3 (75.0-95.3)		.03	
	“Decent” type	75.0 (68.8-87.5)		78.1 (71.9-93.8)		.02	
	“Mature” type	81.3 (71.9-93.8)		75.0 (68.8-87.5)		.04	
Returned the self-test result within 24 hours upon receiving the self-test kit			78.1 (65.6-89.8)		78.1 (71.9-93.8)		.04
**Confidence in self-testing procedures**							
	Collecting sufficient oral fluid^a^	75.0 (68.8-87.5)		81.3 (71.9-93.8)		.001	
	Collecting sufficient blood^a^	75.0 (65.6-87.5)		84.4 (75.0-93.8)		<.001	
	Correctly performing the HIV self-test^a^	75.0 (65.6-84.4)		84.4 (71.9-93.8)		<.001	
	Interpreting the self-test result^a^	75.0 (65.6-84.4)		81.3 (71.9-93.8)		<.001	

^a^Gave a score of 9 or above on a scale of 0 to 10.

Promoters giving at least 80 points in the SUS scale at test result upload (184/351, 52.4%) were more likely to have passed the online training, made a peer referral, used social media for sex networking, used pre-exposure prophylaxis (PrEP) for HIV prevention when having condomless anal intercourse (CLAI) with known male sex partners, and given a higher UI score (*P*<.05). They were also more confident in using the self-test (*P*<.001), while preferring no assistance for the self-test (*P*=.001), and had requested an oral fluid self-test (*P*=.04) (Table 4). While promoters did not give more correct answers in HIV-related knowledge, nonpromoters were more likely to wrongfully consider blood donation an appropriate way for HIV screening in the HIV-related knowledge assessment (odds ratio [OR] 2.57, 95% CI 1.33-4.95; *P*=.01). They also gave a higher SEQ score after the test kit request, result upload, online training, and peer referral (*P*<.01).

**Table 4:** Characteristics of participants giving a System Usability Scale score of 80 or above (promoters) (N=351).

Variable		Promoters (N=184), n (%)	Nonpromoters (N=167), n (%)	OR^a^ (95% CI)	*P* value
Used social media for sex networking in the past 1 year		90 (48.9)	61 (36.5)	1.66 (1.09-2.55)	.02
Used PrEP^b^ in the past 1 year (N=165^c^)		59 (70.2)	40 (49.4)	2.42 (1.28-4.58)	.006
UI^d^ score of 4 or above on a scale of 1-5		174 (94.6)	119 (71.3)	7.02 (3.42-14.42)	<.001
**Confidence in self-test procedures**					
	Collecting sufficient oral fluid^e^	130 (70.7)	89 (53.3)	2.11 (1.36-3.27)	<.001
	Collecting sufficient blood^e^	102 (55.4)	53 (31.7)	2.68 (1.73-4.14)	<.001
	Correctly performing the HIV self-test^e^	139 (75.5)	90 (53.9)	2.64 (1.68-4.16)	<.001
	Interpreting the self-test result^e^	147 (79.9)	97 (58.1)	2.87 (1.79-4.60)	<.001
	Preferring no assistance for the self-test	132 (71.7)	92 (55.1)	2.07 (1.33-3.22)	.001
Requested for an oral fluid self-test		118 (64.1)	89 (53.3)	1.57 (1.02-2.40)	.04
SEQ^f^ ≥6 on a scale of 1-7 at test kit request (N=348)		147 (80.8)	107 (64.5)	2.32 (1.42-3.77)	<.001
SEQ ≥6 on a scale of 1-7 at result upload		177 (96.2)	123 (73.7)	9.05 (3.94-20.75)	<.001
SEQ ≥6 on a scale of 1-7 at online training (N=213)		53 (44.5)	24 (25.5)	2.34 (1.30-4.22)	.004
SEQ ≥6 on a scale of 1-7 at peer referral (N=119)		43 (61.4)	18 (36.7)	2.74 (1.29-5.83)	.008
Passed online training		115 (62.5)	83 (49.7)	1.69 (1.10-2.58)	.02
Made a peer referral		66 (35.9)	43 (25.8)	1.61 (1.02-2.55)	.04

^a^OR: odds ratio.

^b^PrEP: pre-exposure prophylaxis.

^c^Among participants who had condomless anal intercourse with previously acquired male sex partners.

^d^UI: user interface.

^e^Gave a score of 9 or above on a scale of 0 to 10.

^f^SEQ: Single Ease Question.

Participants in the neumorphism arm gave a higher SUS score than those in the gamification arm (median 82.5 vs 77.5; “Excellent” vs “Good” using the adjective rating scale [24]; *P*<.001), and more participants in the neumorphism arm had an acceptance level (*P*<.001) and a promoter level (*P*=.002) (Table 5). The learnability score was similar between the 2 arms; therefore, the differences in the scores of SUS items 4 and 10 were insignificant. On the other hand, the usability score was higher in the neumorphism arm (*P*<.001). The proportions of participants who were satisfied with the UI (*P*=.01) and agreed to all positively worded statements in the SUS, except for item 9 on confidence in system use, were higher in the neumorphism arm (*P*<.01).

**Table 5:** Comparison of individual and overall System Usability Scale items, learnability scores, and usability scores between the gamification and neumorphism arms (N=351).

Statement/variable	Gamification arm (N=176), median (IQR)	Neumorphism arm (N=175), median (IQR)	*P* value	Gamification arm (N=176), n (%)^a^	Neumorphism arm (N=175), n (%)^a^	OR^b^ (95% CI)	*P* value
I am satisfied with the system interface design	4 (4-5)	4 (4-5)	.01	138 (78.4)	155 (88.6)	0.47 (0.26-0.84)	.01
SUS^c^ #1: I think that I would like to use this system frequently	4 (4-5)	4 (4-5)	.007	136 (77.3)	158 (90.3)	0.37 (0.20-0.67)	.01
SUS #2: I found the system unnecessarily complex	2 (1-3)	2 (1-2)	.01	23 (13.1)	17 (9.7)	1.40 (0.72-2.72)	.32
SUS #3: I thought the system was easy to use	4 (4-5)	4 (4-5)	.008	145 (82.4)	162 (92.6)	0.38 (0.19-0.74)	.004
SUS #4: I think that I would need the support of a technical person to be able to use this system	1 (1-2)	1 (1-2)	.40	14 (8.0)	9 (5.1)	1.59 (0.67-3.79)	.29
SUS #5: I found the various functions in this system were well integrated	4 (3-4)	4 (4-5)	<.001	103 (58.5)	145 (82.9)	0.29 (0.18-0.48)	<.001
SUS #6: I thought there was too much inconsistency in this system	2 (1-3)	1 (1-2)	<.001	9 (5.1)	8 (4.6)	1.13 (0.42-2.99)	.81
SUS #7: I would imagine that most people would learn to use this system very quickly	4 (4-5)	4 (4-5)	.005	137 (77.8)	156 (89.1)	0.43 (0.24-0.78)	.004
SUS #8: I found the system very awkward to use	2 (1-2)	1 (1-2)	.002	9 (5.1)	9 (5.1)	0.99 (0.39-2.57)	.99
SUS #9: I felt very confident using the system	4 (4-5)	4 (4-5)	.09	149 (84.7)	159 (90.9)	0.56 (0.29-1.07)	.08
SUS #10: I needed to learn a lot of things before I could get going with this system	2 (1-3)	2 (1-2)	.06	15 (8.5)	14 (8.0)	1.07 (0.50-2.29)	.86
SUS acceptance level (≥71)	N/A^d^	N/A	N/A	109 (61.9)	147 (84.0)	0.31 (0.19-0.51)	<.001
SUS promoter level (≥80)	N/A	N/A	N/A	78 (44.3)	106 (60.6)	0.52 (0.34-0.79)	.002
SUS overall score	77.5 (65.6-89.4)	82.5 (75.0-92.5)	<.001	N/A	N/A	N/A	N/A
SUS learnability score	87.5 (62.5-100.0)	87.5 (75.0-100.0)	.11	N/A	N/A	N/A	N/A
SUS usability score	75.0 (65.6-87.5)	84.4 (75.0-93.8)	<.001	N/A	N/A	N/A	N/A

^a^Percentage refers to the proportion of participants giving a score of 4 or above on a scale of 5 for the respective statement.

^b^OR: odds ratio.

^c^SUS: System Usability Scale.

^d^N/A: not applicable.

**Figure 3:**
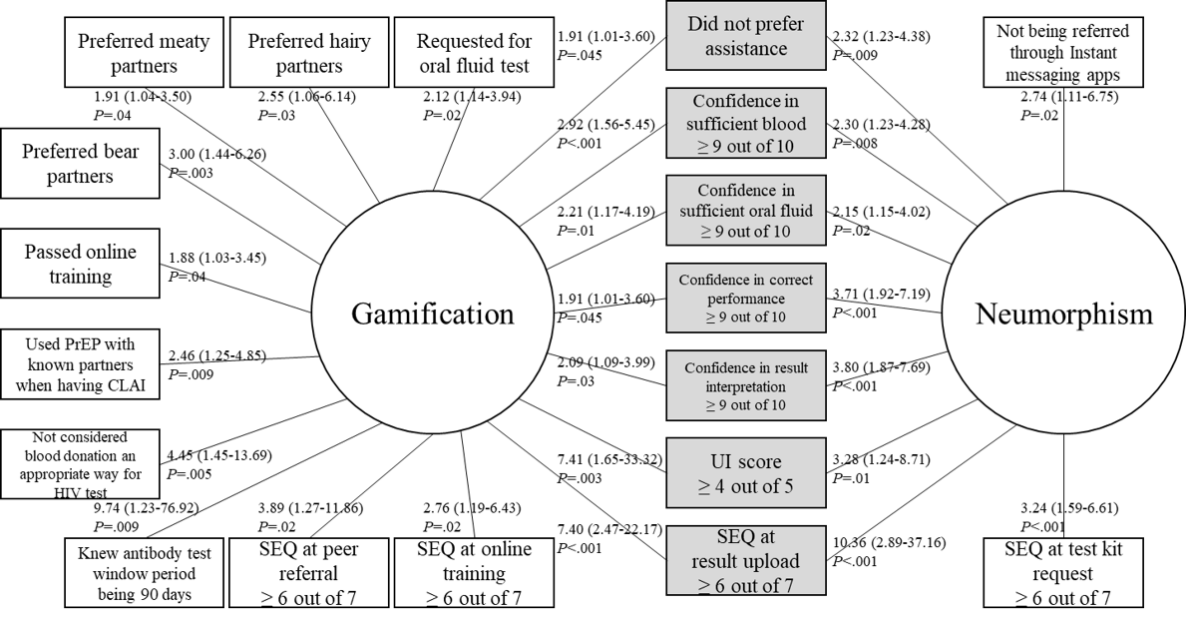
Factors associated with promoters in the gamification and neumorphism arms. Shaded variables are mutually associated with both arms. Values on the link are odds ratios (95% CIs). CLAI: condomless anal intercourse; PrEP: pre-exposure prophylaxis; SEQ: single ease question; UI: user interface.

To identify the distinct characteristics associated with a promoter-level SUS score with gamified and neumorphic designs, subgroup analyses were performed (Figure 3). Promoters in the gamification arm were more likely to prefer sex partners to be of “meaty,” “hairy,” or “bear” body image types, request for an oral fluid test, and give a higher SEQ score at peer referral (*P*<.05). They were also less likely to give a wrong answer in some HIV-related knowledge questions (*P*<.05) and more likely to use PrEP when having CLAI with known sex partners (*P*=.009) and pass the online training (*P*=.04). Neumorphism arm promoters were more likely to give a higher SEQ score at test kit request (*P*<.001) and be referred from channels other than instant messaging apps (*P*=.02). The multivariable logistic regression model showed that, other than high UI (*P*<.001) and SEQ (*P*=.02) scores, promoters in the gamification arm were more likely to request for an oral fluid test (*P*=.001), prefer a “bear” body image type partner (*P*=.02), and be confident in collecting blood samples (*P*=.003), while those in the neumorphism arm gave a higher SEQ score at result upload (*P*<.001) and were confident in performing the self-test (*P*<.001).

## Discussion

The social network–based HIV self-test web application was well-accepted by MSM, with an overall median SUS score of 80. Higher system usability was associated with confidence in using the self-test kit and completing the self-test quicker, indicating that this application had linked the user experiences of online activities with offline ones. MSM encountering issues in learning to use the system were less knowledgeable about HIV and more likely to be bisexual. This echoed the results from a previous study showing that bisexual men had lower HIV-related knowledge, who had a higher level of stigma or discrimination associated with the infection [26]. Online social networking tools could be leveraged to promote health behaviors [27], and users gave higher usability and learnability scores. Together with the participants’ distinct preferences in sex partners’ characteristics, our results informed the need of addressing bisexual MSM and the choice of key opinion leaders of different body image types in future HIV-related promotion campaigns through social media.

The study results confirmed that participants who were satisfied with the system and with a high perceived ease of use throughout the engagement, were more likely to promote it to their peers [21]. Promoters were confident in using the self-test without assistance. They tended to have used PrEP for HIV prevention and possessed adequate HIV knowledge; therefore, they were competent to share their positive self-test experiences and knowledge in HIV, and access to HIV prevention services with their peers, which could in turn invite them to get self-tested. On the other hand, knowledge deficit may have hindered some from referring their peers to engage in prevention programs. This showed that a satisfactory UX, adequate HIV knowledge, and peer referral and support could facilitate HIV testing promotion programs [28].

Participants in the 2 arms were satisfied with the system and the interface design. Compared with the findings in the gamification arm, higher SUS and UI scores were observed among participants assigned to the neumorphism arm, with over half giving a promoter-level score. The impact of UI on UX was only reflected in the usability facet, but not the learnability facet. In reference to the 10 usability heuristics of UI design [29], “esthetic and minimalist design” was found to be of particular importance in contrasting the 2 designs in this study. Neumorphism adopts a simplistic visual design conforming with the heuristics, whereas the colorful graphics in the gamification interface may have distracted users from the primary goal and subsequently weakened its usability. Nevertheless, no matter which arm participants were assigned to, almost all users considered the system consistent and agreed that they did not need to learn a lot of things or ask for support to use the system. These were underpinned by the fourth and sixth heuristics, which were “consistency and standards” and “recognition rather than recall,” respectively.

Preferences in UI design style may be related to one’s personality, age, gender, and education [30,31]. Shared characteristics among promoters in the 2 arms included higher SEQ and UI scores at the same time when the promoter-level SUS score was given, and confidence in performing the self-test without assistance. No personal attributes were associated with promoters in the neumorphism arm, but those in the gamification arm were featured with partner body image type preference, PrEP use, and HIV-related knowledge. This highlighted that the gamified UI was favored by selected MSM, whereas the neumorphic design was more universally accepted. Our finding provides an insight into the future selection of art styles for designing promotion materials. Although physical appearance traits may differ contextually, a previous study in the United States has suggested that the bear community could be a unique group characterized by body image type in the MSM population, which may be at higher HIV risk than others [32]. If a campaign is launched to target them and their potential partners, a gamified approach could be of use. A previous study also showed that gamification could make HIV and sexually transmitted infection testing less stressful [33]. On the contrary, a neumorphic style would be more suitable to reach the general MSM community.

This study has several limitations. First, the use of sensitive sexual behavioral questions might have given rise to social desirability bias in responders, which we have minimized by adopting a self-administered approach. We also used a short recall period to reduce recall bias. Second, in a randomized parallel-group design, we were unable to capture participants’ preferences by presenting them with both UI designs. Instead, we compared the scores between 2 arms and conducted subgroup analyses to identify associating factors with scores reaching the promoter level. The SUS scores used in this study were collected at test result upload; therefore, all participants who did not upload their results were excluded. As the primary goal of the study was to have participants refer their peers for self-testing, uploading a test result was a necessary step, and thus, only those who had uploaded a test result were considered to have completed the study procedure and were included in the analyses. Third, HIV knowledge was not assessed with a conventional validated scale, as such a scale may have been outdated with the recent development of HIV medicine and PrEP. The knowledge questionnaire we designed could however only serve the purpose of identifying a knowledge gap in the community, which was relevant to the theme of the study. Fourth, generalization to other settings with different cultural backgrounds could be limited, particularly for body image types. Finally, neumorphism has an inherent weakness in accessibility due to the lack of contrast in color. We did not enquire about users’ color vision; hence, we were unable to consider the impact of color vision deficiency on the usability of the 2 UI designs.

In conclusion, we demonstrated the effective use of a web application that could link users from online activities to offline engagement for the purpose of promoting HIV testing through the collection of self-test kits. The experience of the offline activity could impact subsequent online engagements, which involve peer referral for extending the network of MSM who could go for HIV self-testing as a health promotion strategy. We also identified MSM’s preferences in and possible implementation scenarios of gamified and neumorphic UI designs, laying the scientific foundation for future UI and UX designs for internet interventions targeting the MSM community.

## Appendix

Multimedia Appendix 1 Screenshots of the pages in both the gamification and neumorphism arms.

Multimedia Appendix 2 CONSORT-eHEALTH checklist (V 1.6.1).

## Abbreviations

CLAI: condomless anal intercourse
MSM: men who have sex with men
OR: odds ratio
PrEP: pre-exposure prophylaxis
SEQ: Single Ease Question
SUS: System Usability Scale
UI: user interface
UX: user experience
