# Supplementary material for: User Experience and Usability of Neumorphism and Gamification User Interface Designs in an HIV Self-Test Referral Program for Men Who Have Sex With Men: Prospective Open-Label Parallel-Group Randomized Controlled Trial
Source: JMIR Serious Games. 2022 Jun 22;10(2):e35869. doi: 10.2196/35869 (PMC9260534; doi:10.2196/35869)

Questionnaire page (gamification arm)

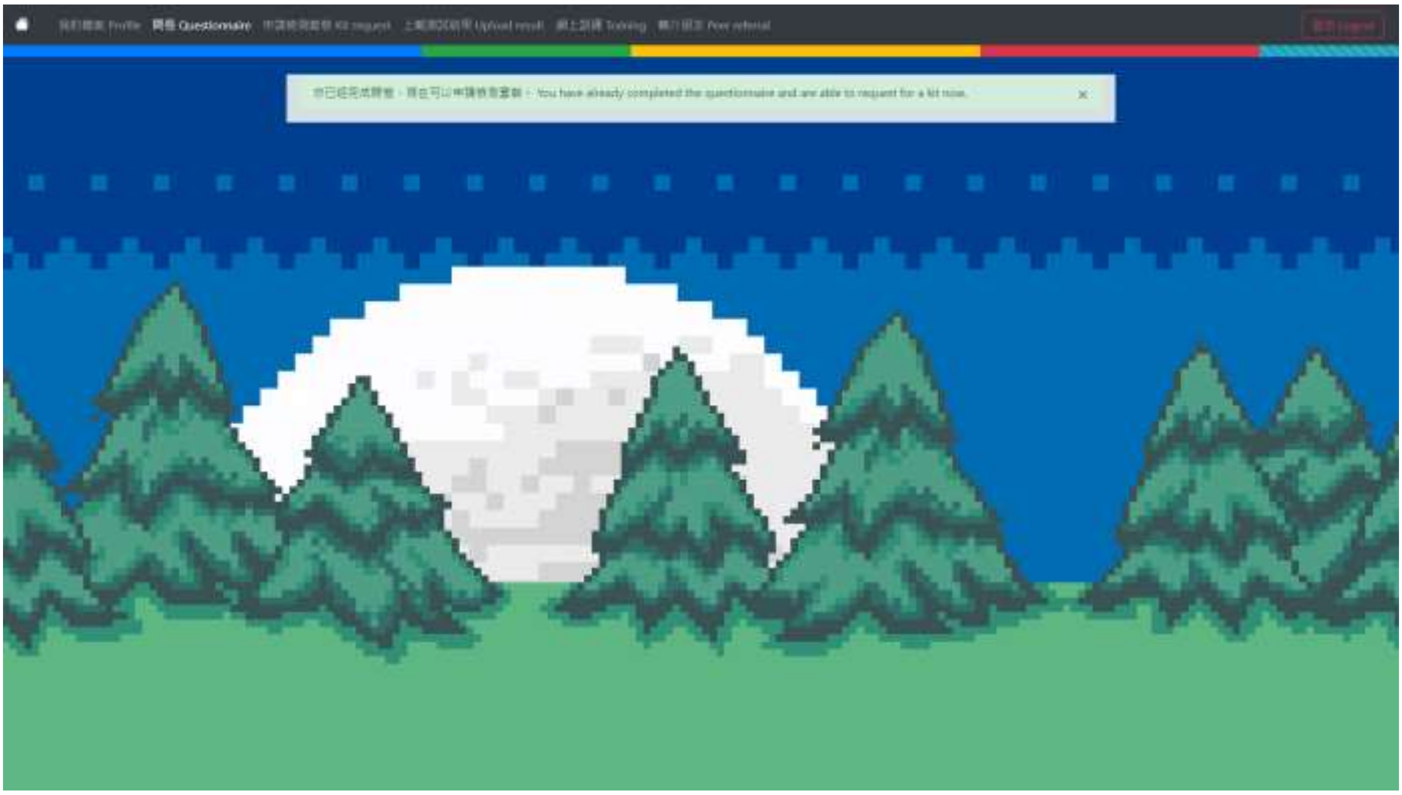

Questionnaire page (neumorphism arm)

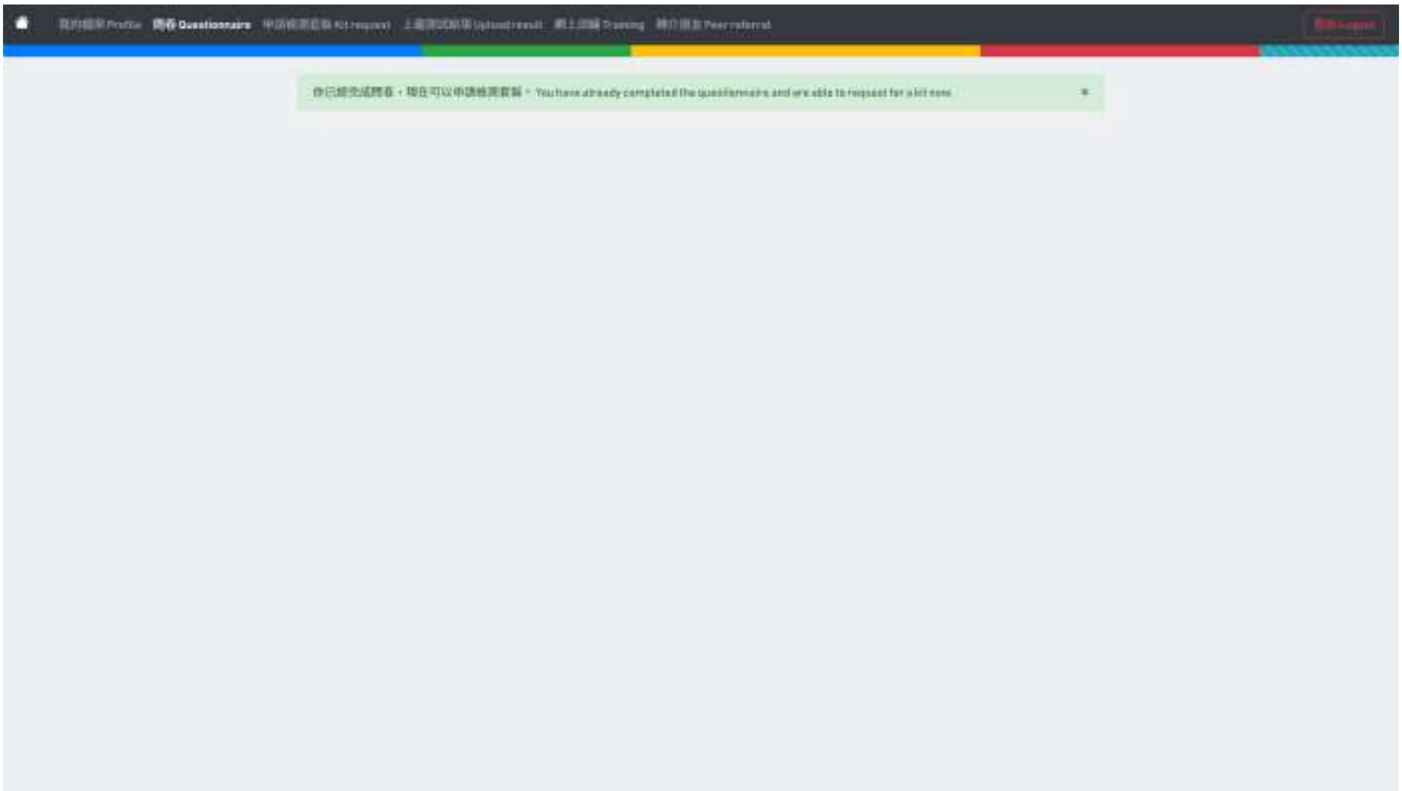

Kit request page (gamification arm)

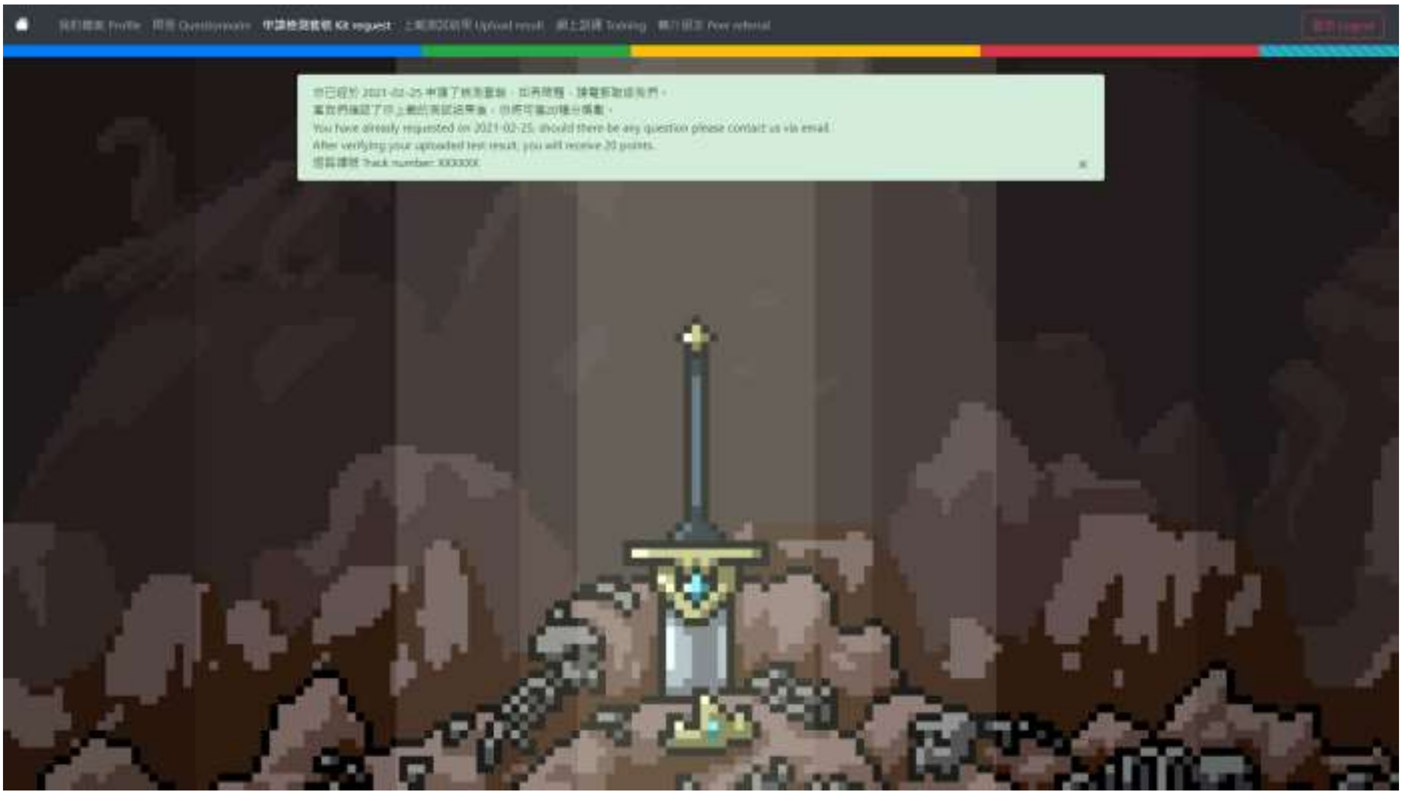

Kit request page (neumorphism arm)

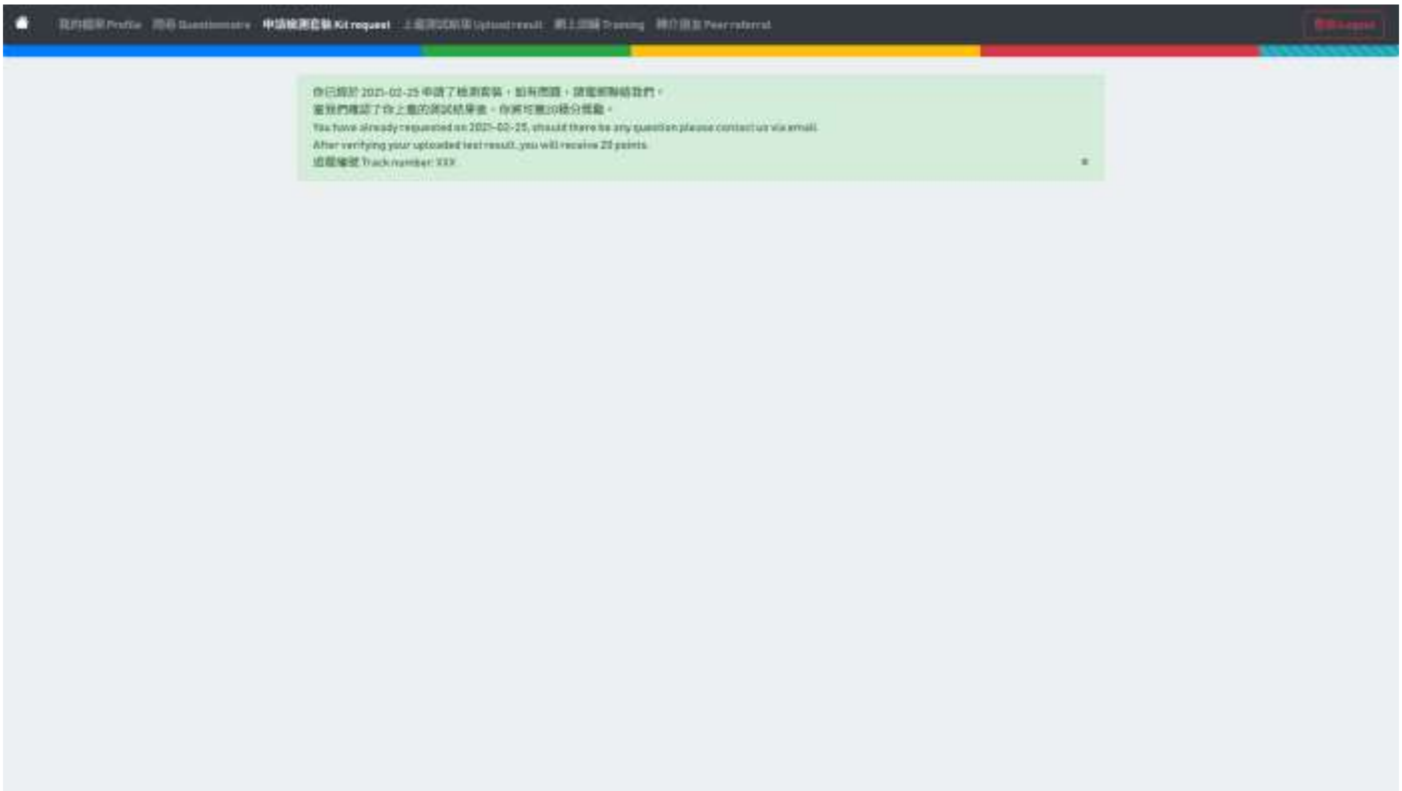

Result upload page (gamification arm)

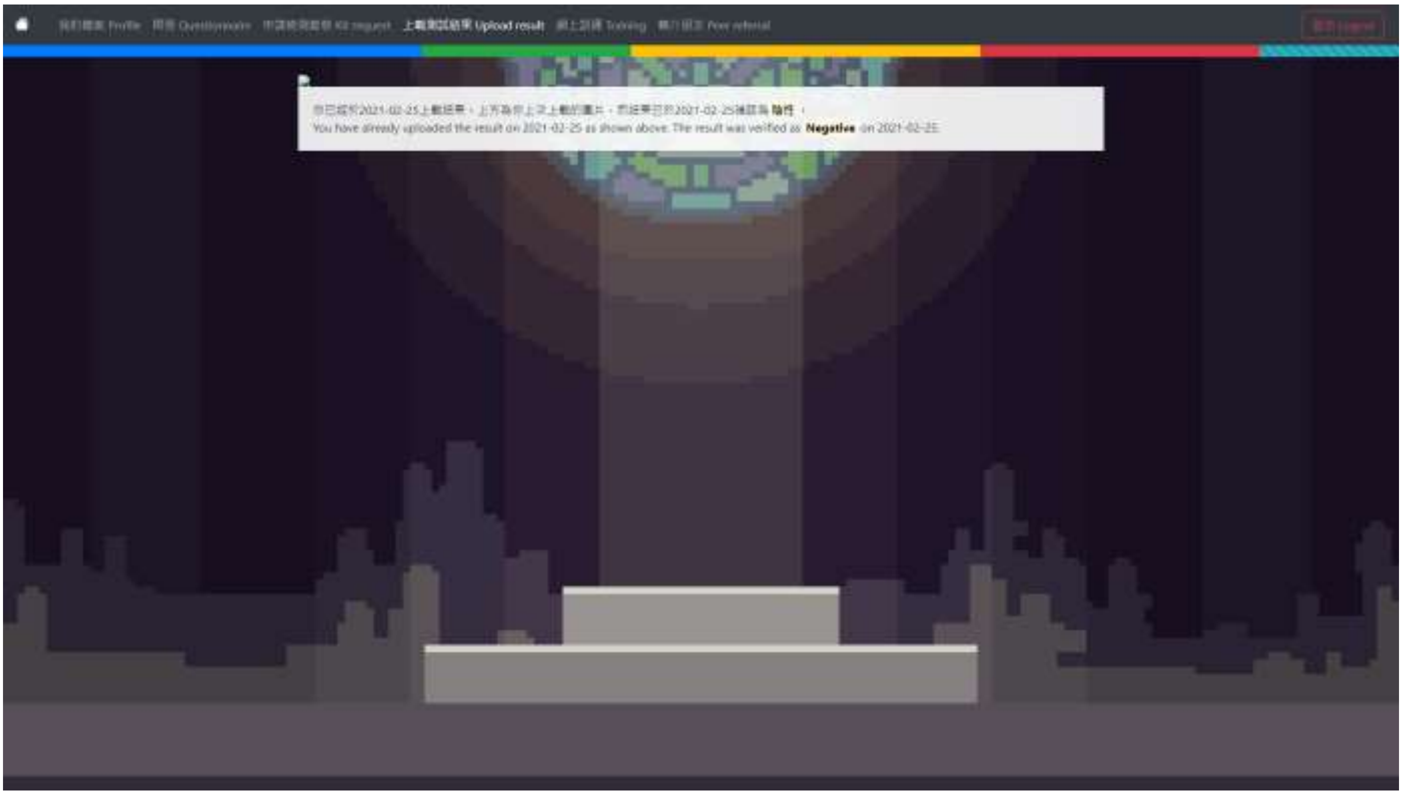

Result upload page (neumorphism arm)

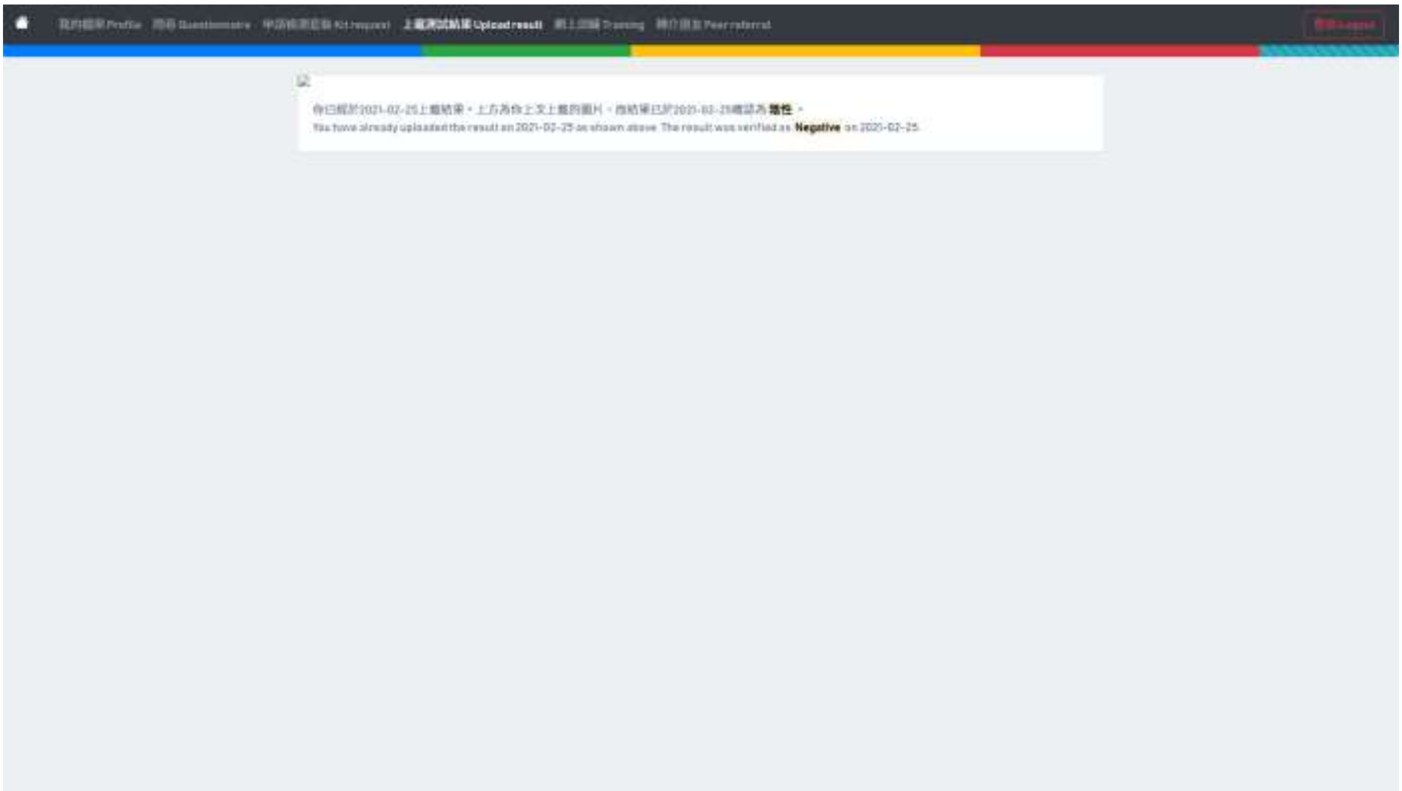

Training page (gamification arm)

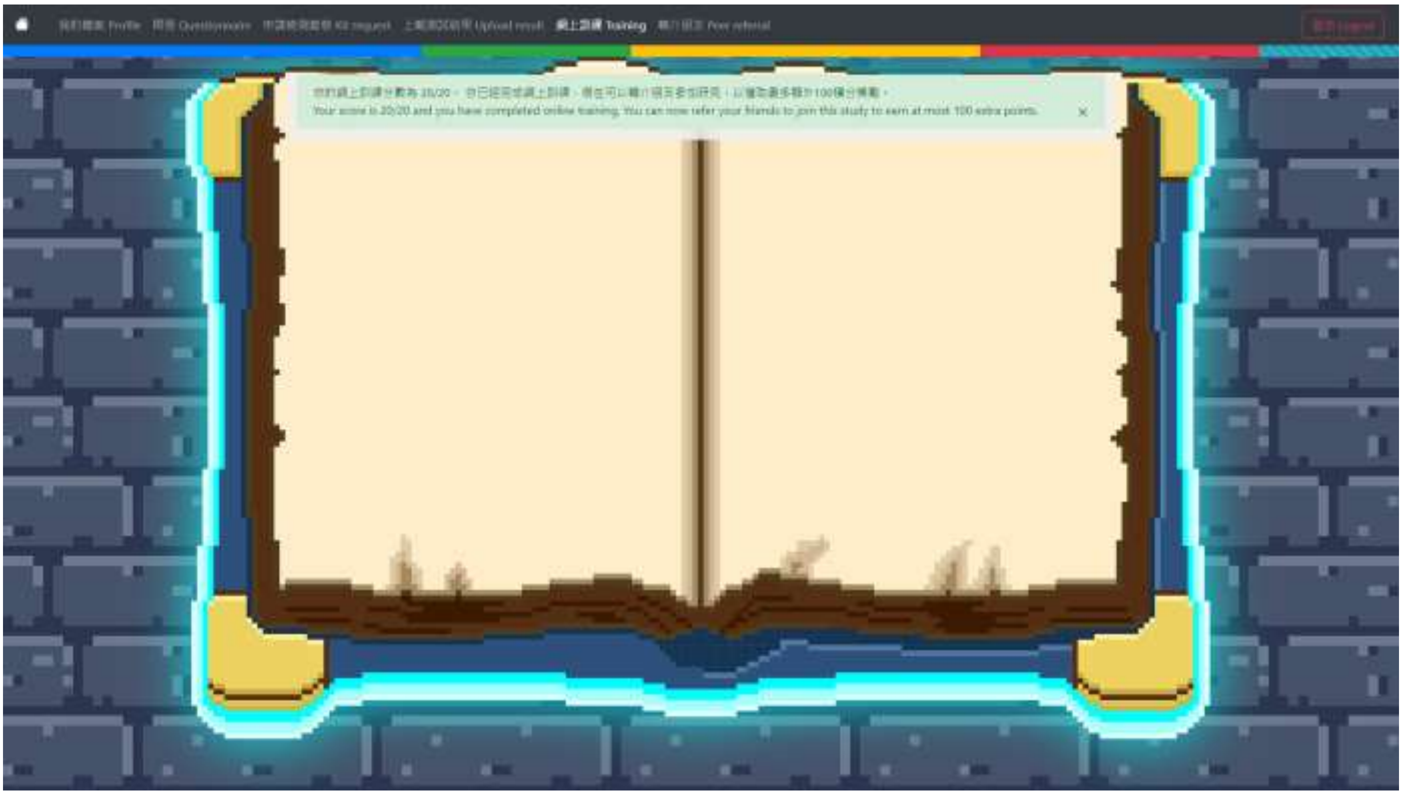

Training page (neumorphism arm)

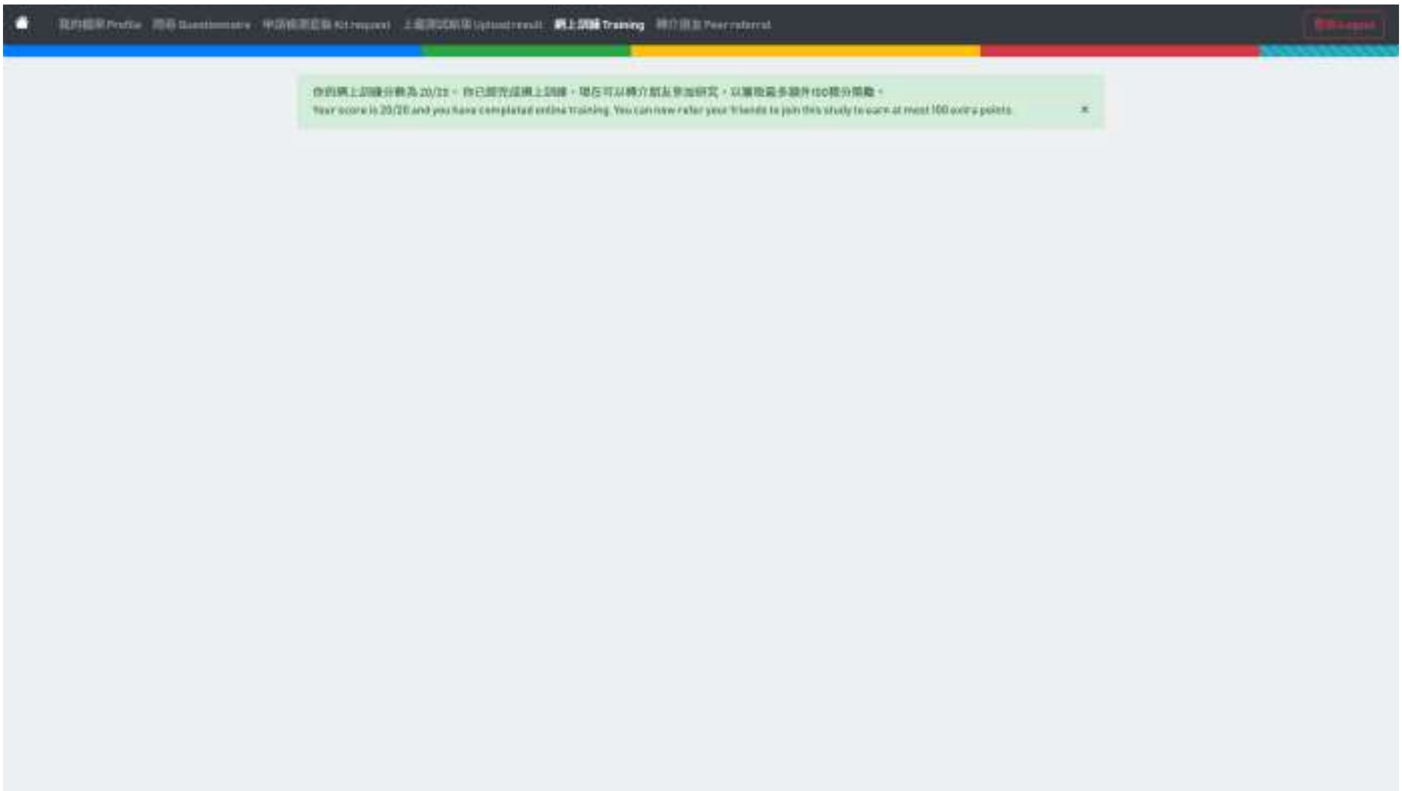

Peer referral page (gamification arm)

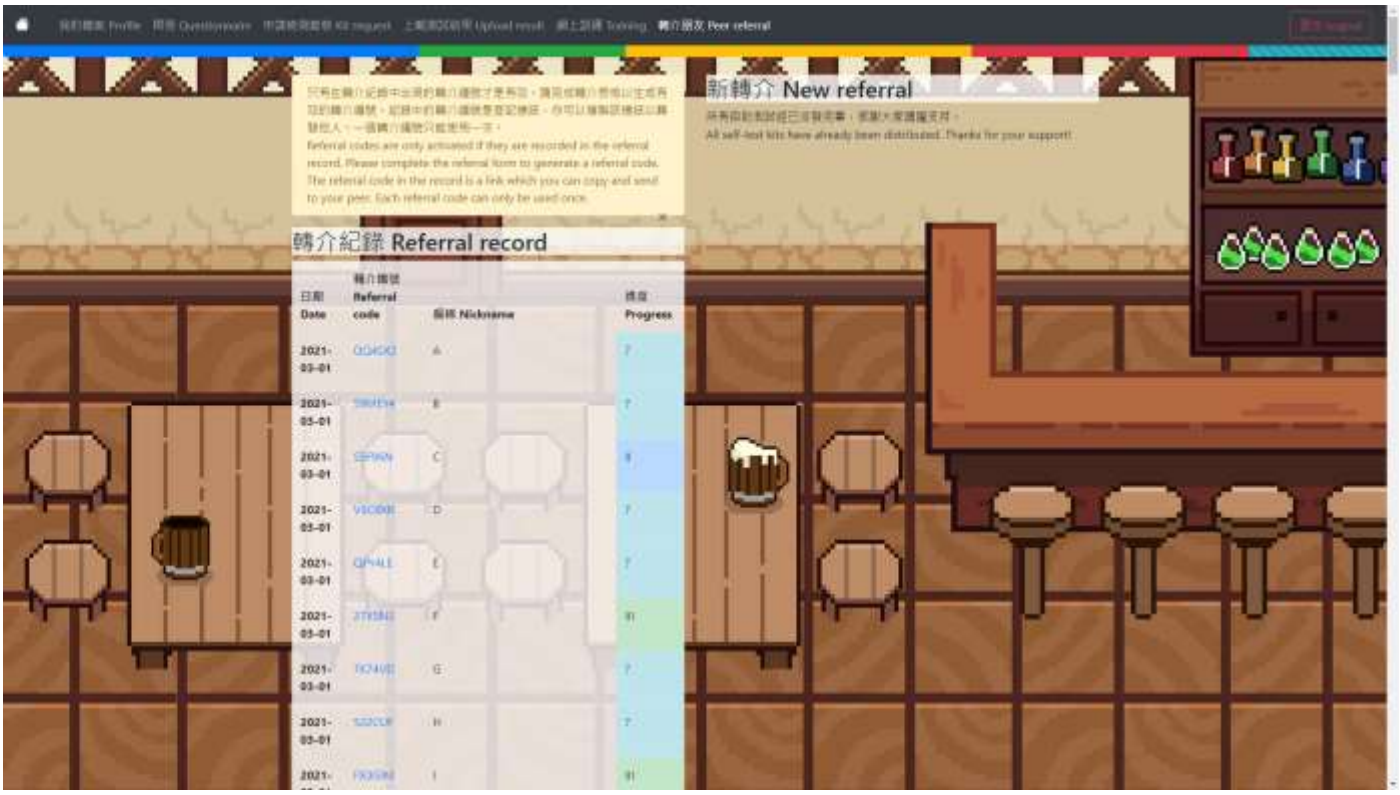

Peer referral page (neumorphism arm)

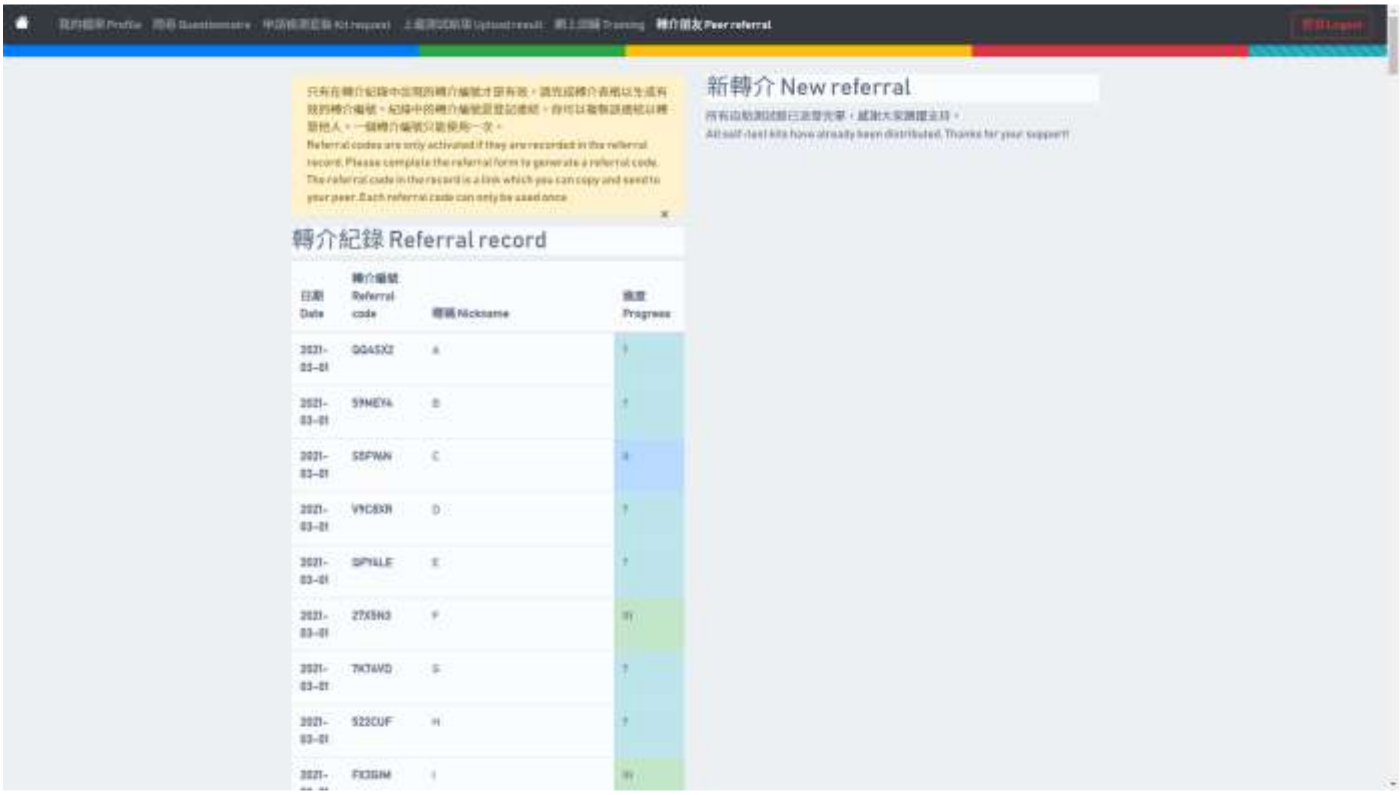

Redemption page (gamification arm)

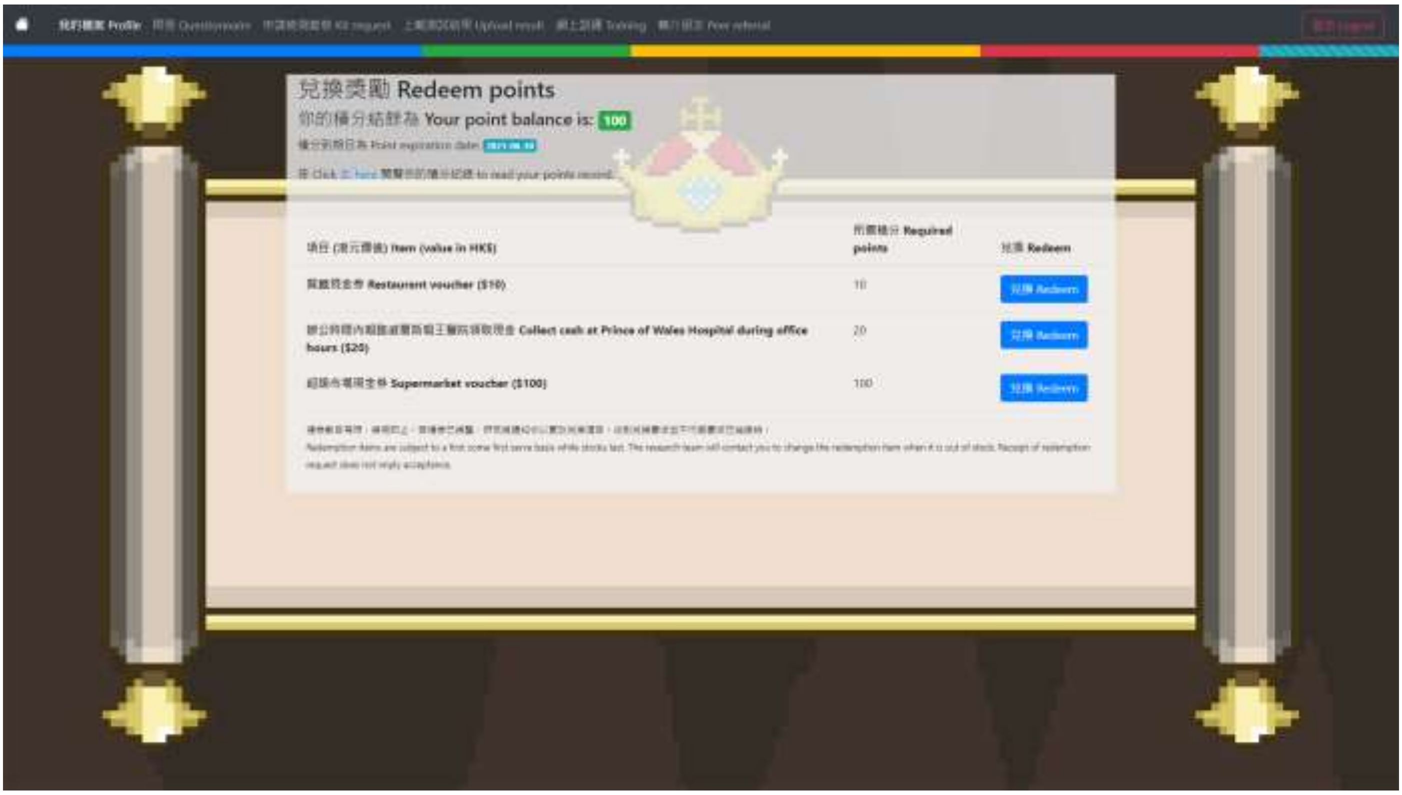

Redemption page (neumorphism arm)

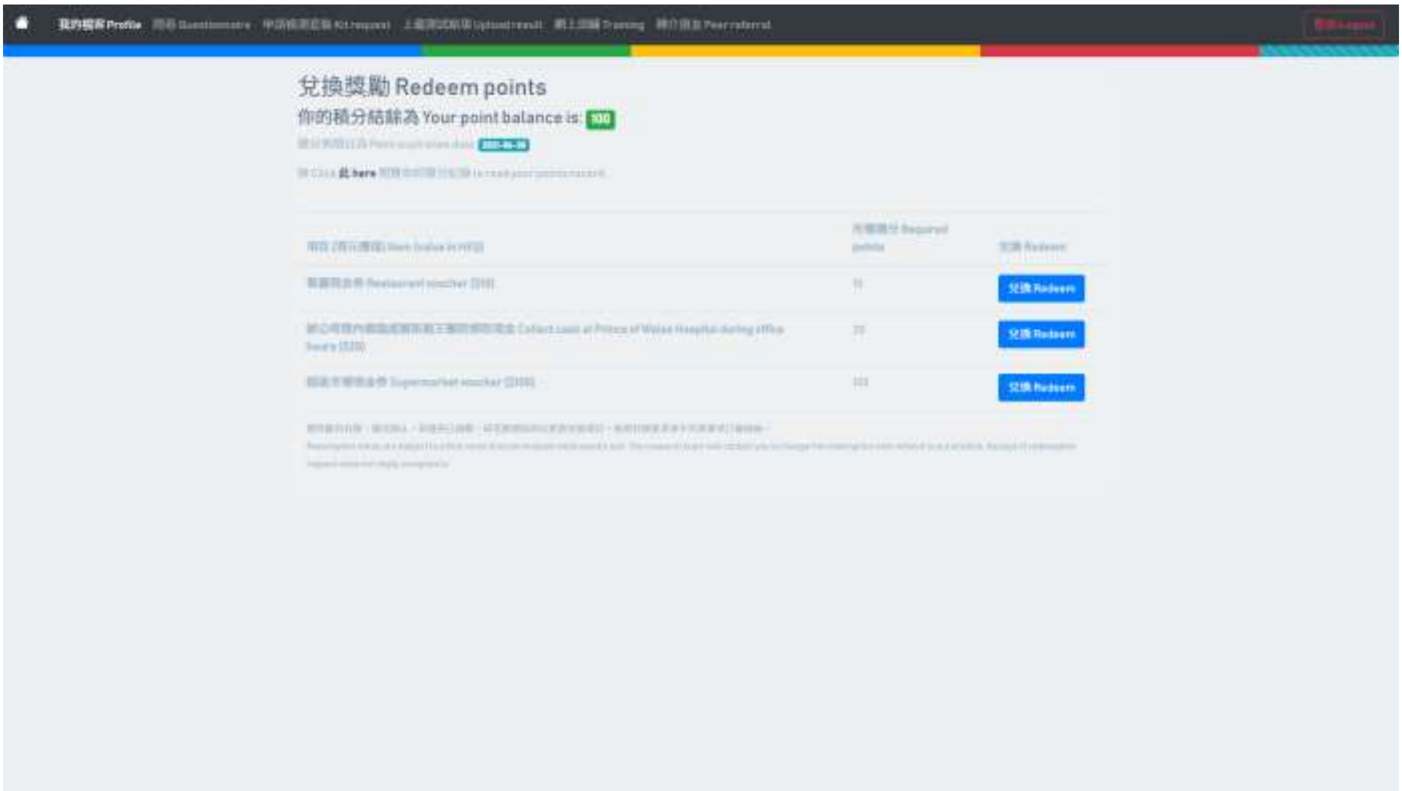

Introduction page (neumorphism)

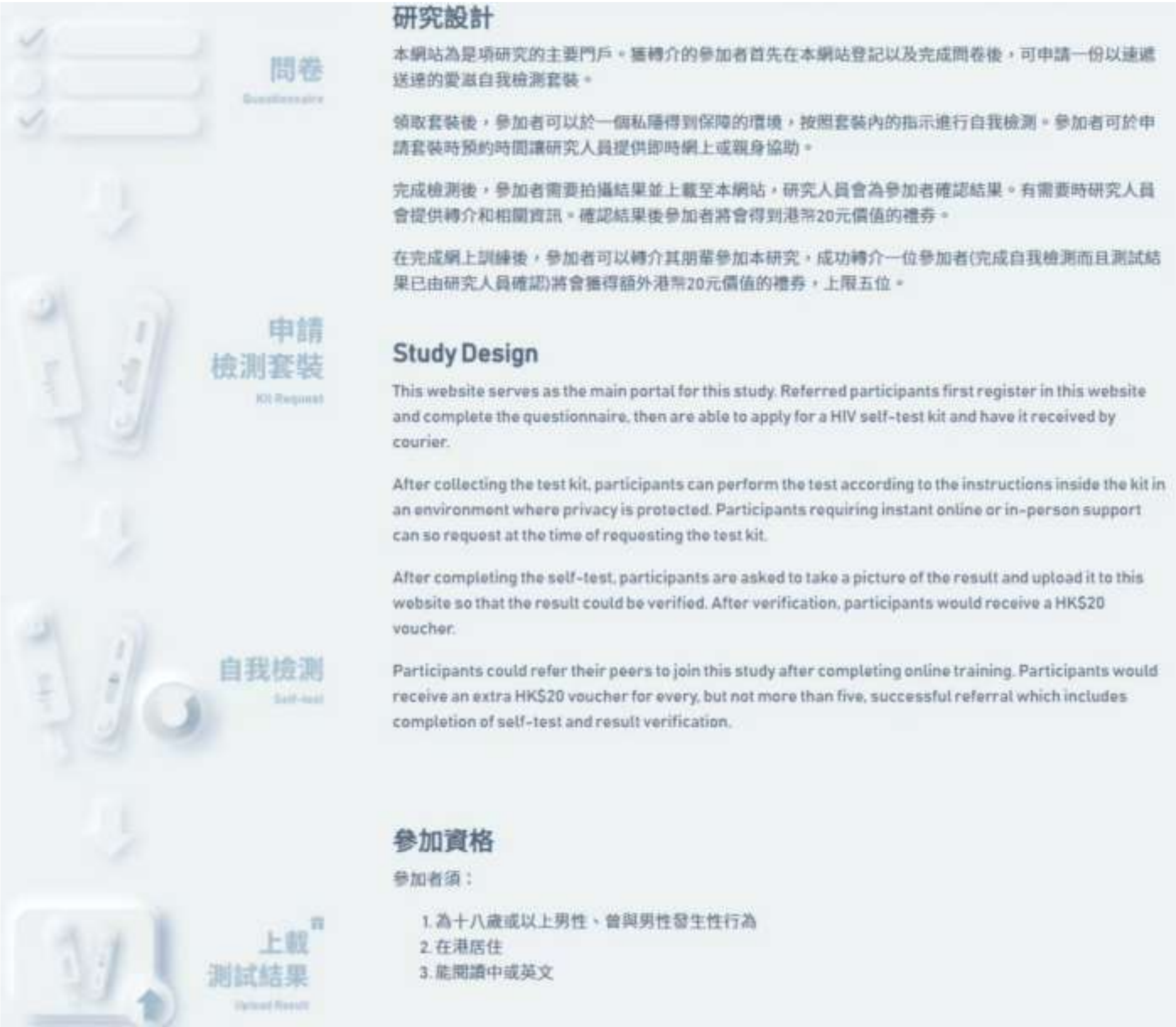

Example of radio buttons in the neumorphism arm (training page)

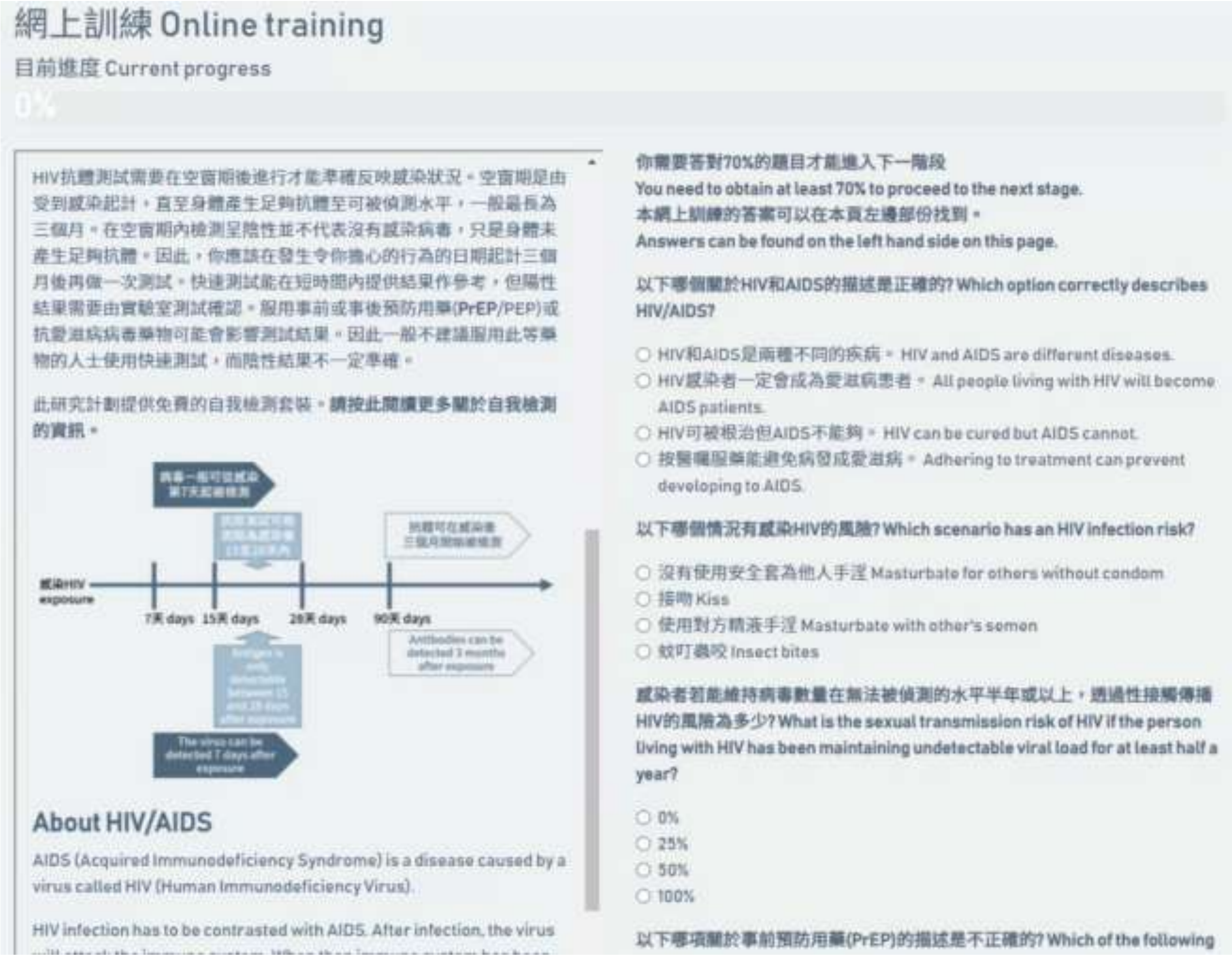

Example of checkboxes and drop-down lists in the neumorphism arm (questionnaire page)

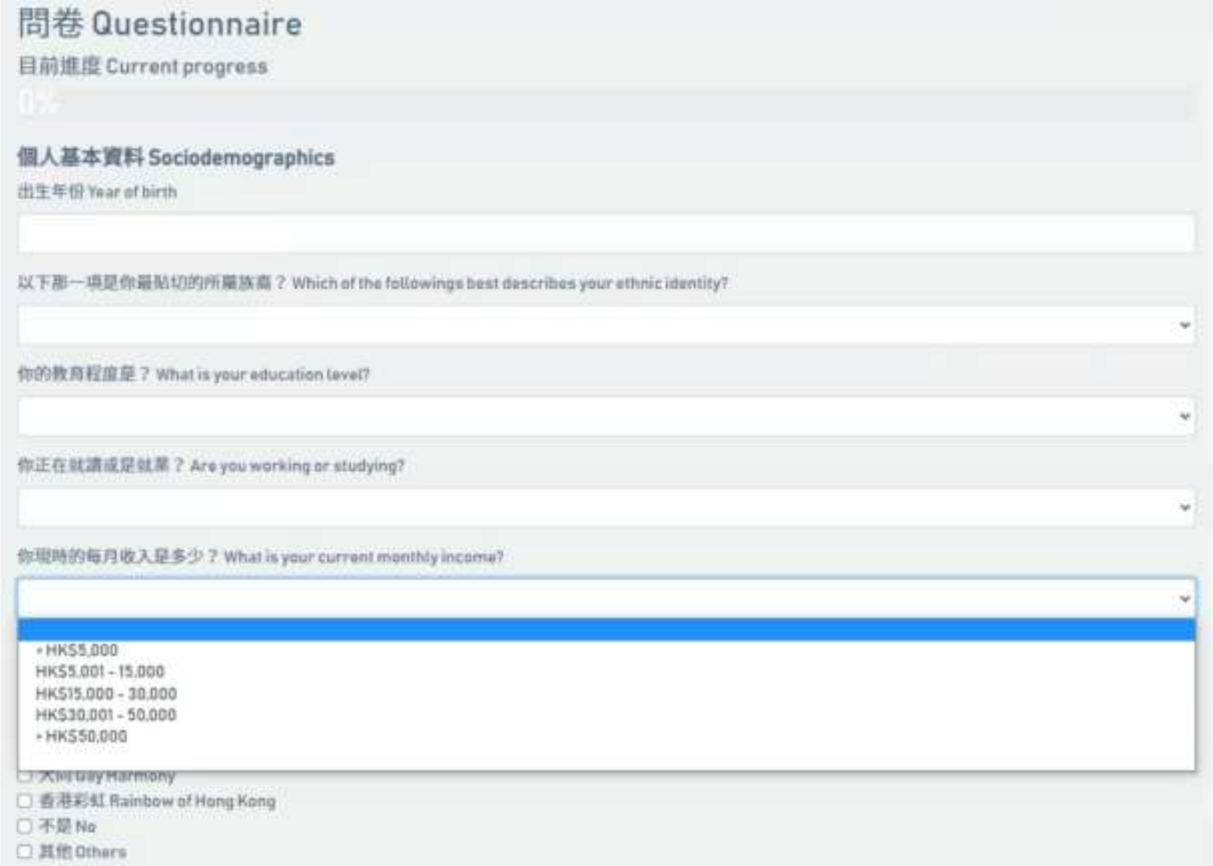

Supplement: Multimedia Appendix 1 [file games_v10i2e35869_app1.pdf]
